# Pamphlets

**Published:** 1870-09

**Authors:** 


### Pamphlets.

*A Guide to the Examination of the Urine. For the Practitioner and Student.* By J. WICKHAM LEGG, M.D., M.R.C.P., Physician to the St. George, Hanover Square, Dispensary. Second edition. Philadelphia: Lindsay & Blakiston. 1870. Pp. 89.

This is a little manual with flexible covers, containing a succinct statement of the more important characters of the urine and how to determine them, with an appendix describing the manner of estimating the urea, chlorides, phosphates, sugar, etc., by volumetric or other rapid analysis.

*Sun Stroke and its Theory.* By ELY VAN DE WARKER, M.D., Syracuse, N. Y. Reprinted from the N. Y. Med. Jour. D. Appleton & Co. 1870. Pp. 24.

The author discusses the history and statistics, diagnosis, prognosis, theory, pathology, treatment and prophylaxis. He has collected in small space much valuable material. He recommends the cold douche carefully employed so as not to produce too profound sedation. If the patient can swallow, teaspoonful doses of brandy and chloroform, or other diffusible stimulant. Sinapisms, etc., to combat insensibility or sedation. Purgative enemata, followed by an enema of turpentine. Venesection is not commended, and in the convulsive form the douche should not be used. Chloroform in these cases has been advised, but in some cases seemed to hasten the fatal result. Warm baths with mustard, and the free administration of brom. pot. are suggested. Spirit drinkers and those who expose the neck or wear dark clothing are the most liable to be attacked.

Sir Charles Napier, when serving in Sindh, says of an attack of insolation he suffered there: "I had hardly written the above sentence, ten days ago, when I tumbled over by the heat with apoplexy; forty-three others were struck, all Europeans, and all died within three hours except myself! I do not drink! That is the secret. The sun had no ally of liquor among my brains." Light clothing, the head and neck protected from the direct solar rays—better by a dampened handkerchief or even leaves under the head covering. "The only head dress worn by the natives of Abyssinia is a lump of butter, which by melting runs down the neck and shoulders and prevents their drying in the intense heat."

When the case presents itself, it is indispensable for correct prognosis and treatment that its individual peculiarities should be thoroughly appreciated. Dr. Van De Warker has done well in pointing out a number of essentially diverse conditions usually confounded under a common designation.

*Remarks on a New Medium for Preserving and Putting up Pathological and Anatomical Specimens.* Invented by Edward Clapham, M.D., etc., Professor General and Micros. Anat., Medical Dep. Iowa State University. Also, by the same author: *The Introduction and Use of Arsenite of Soda, as a powerful and advantageous Antiseptic, in the Preparation of Cadavera for purposes of Practical Anatomy.* Pp. 12.

Prof. Clapham explains the advantages of the ordinary benzole of commerce, over alcohol, glycerine, etc., in the preservation of soft preparations, in that it does not become discolored by contact with the animal fluids, or cause shreddiness or disintegration of them. It remains free from turbidity however much debris may subside. It does not bleach. Its great brilliancy and high refracting power endow it with superior advantages for displaying dissections of great delicacy and minuteness. Its trivial cost (about one-eighth that of alcohol), and resistance of low temperature where dilute alcohol speedily congeals. In the pamphlet directions are given for its use. Prof. Clapham claims priority in the introduction of this agent for this purpose.

The appendix, on the use of arsenite of soda, contrasts the use of this agent with other antiseptics commonly employed, and gives general directions for its preparation and application. We hope to hear further from this accomplished chemist and anatomist.

*Some Clinical Observations on the Malignant Diseases of the Uterus.* By FORDYCE BARKER, M.D. Read before the N. Y. Academy of Medicine, Feb. 17, 1870. Pp. 32.

Dr. Barker is an indefatigable worker, and observes with a good brain back of his eyes. It is true, scarcely any body else has as good luck as he has in the treatment of pseudo-membranous croup with turpeth mineral, but we can readily overlook this drawback in the light of his varied and excellent contributions to the progress of medical science and art. In the present monograph he does not discuss the pathology of the malignant diseases of the uterus, other members of the Academy having recently covered the ground.

Under the head of Frequency of the Disease, he inclines to the opinion that it is, relatively, as often met with in the country as the city. From the tables of the Registrar General, it appears there were in England from 1838 to 1842, 3,000 deaths from cancer of the uterus. In France, in the department of the Seine, from (and inclusive of) the year 1830 to 1835, there were 2,480 deaths from the same cause. From the mortuary records of New York city, there were 431 deaths from cancer of the uterus in the ten years ending Dec. 31st, 1865. The deaths during the three succeeding years and the first nine months of 1869, were 298. Comparative statistics show the Parisian mortality from this cause to exceed that of New York in the proportion of 413 to 79 annually in the years compared.

It would appear that the African race are comparatively exempt from the disease. With regard to the *Etiology* we deem Prof. Barker's remarks eminently worthy of quotation:

"Now, to my mind, all that these statistics prove is, that a vitiated constitution—a diathesis, whether it be inherited or acquired, whether it be tuberculous or cancerous, may predispose to the development of cancer. All the other causes which authors have assigned as predisposing to this disease, amount to just this and no more. I should say, then, nothing is known in regard to the *etiology* of this disease, as science, up to the present time, utterly fails to explain what are the causes of this special form of heteromorphous development. I shall add, there is no evidence that the *disease* is, in any proper use of the term, *hereditary*. The *diathesis* is, undoubtedly, often inherited. But it does not follow that the child of a parent who has died from cancer necessarily or even generally inherits the diathesis; for both the diathesis and the disease of the parent may have been acquired subsequent to the conception and birth of the child."

Not only this, but it should be remembered that the child has *two* parents, and peculiarities of organization result from this conjoint origin which may give the child a "constitution" or "diathesis" totally diverse from those of the parents—crudely expressed in "crossing of the breeds" in animals. The tendency towards death or disease in individuals, is usually better observed in brothers and sisters than in parents and children. One of the profoundest truths in etiology is contained right here. The *diathesis*, which in a parent may ultimate in tuberculous or cancerous cachexiæ, in diabetes, Bright's disease, paralysis, insanity, or inveterate cutaneous eruption, etc., etc., etc., by the controlling influence of joint parentage, climatic, dietetic, and, indeed, any and all other influences capable of affecting the body or its parts in anywise, may show its effect in a manner totally dissimilar in the child. There is a sense in which all diseases are hereditary—hereditary as tribal and national resemblances.

Dr. Barker's record of the comparative frequency of the forms of malignant disease of the uterus is as follows:

| | |
|---|---|
| Cancer of the uterus, - - - - - | 487 |
| Cauliflower excrescence, - - - - - | 18 |
| Corroding ulcer, - - - - - - | 6 |
| Recurrent fibroid, - - - - - - | 3 |

Scirrhus is "one of the rarest" forms, and colloid "very rare." He accepts the distinction between cancer and cancroid: the latter, or locally malignant form, is often extirpated and does not recur; the former is cured, if ever, so infrequently that the cases may be recorded as exceptional.

The monograph does not detail the symptoms systematically, as other authors sufficiently indicate them. Vaginal discharge, pain and hemorrhage are the three most prominent and characteristic symptoms. Primary symptoms are usually slight. None of the vaginal discharges are pathognomonic. Contrary to our own idea he asserts there is nothing peculiar or characteristic in their odor. In his experience pain rarely occurs as a prominent symptom until the disease is far advanced. In a large number of cases he has been struck by the absence of all appearance of cachexia up to the time of death. Peritonitis occurred in a large proportion of cases. Disorders of the urinary apparatus, etc., ordinarily only in the advanced stages.

Credit is given to Dr. Henry Bennett, of London, for the best exposition of the diagnosis of malignant disease of the cervix, and to Sir James Simpson for that when its primary seat is the mucous membrane of the body, and to Grailly Hewitt for that which is primarily interstitial in its seat.

The author observes: "In cases where vaginal examination fails to detect disease of the cervix, I regard hemorrhage, coming on spontaneously some years after the climacteric, as almost conclusive evidence of malignant disease, for I have never known polypi or fibrous tumors to bleed, for the first time, any considerable period after menstruation has ceased."

As to the duration of the disease, Prof. B. says his patients have almost invariably outlived his expectations as based on the statements of most authors. In 26 cases under his own observation the average duration was three years and six months. In one case the patient is still living although the diagnosis is unmistakable, the cervix gone, etc., although it was made out eleven years since.

Notwithstanding the despondent impression which the young practitioner is likely to get from the systematic treatises on gynæcology, the author assures the reader that much can be done in the way of palliation and prolongation of life. "We can certainly improve nutrition, relieve pain, secure sleep, and arrest the offensive sanious discharges and hemorrhage, and thus notably prolong life and make it tolerable to the end."

For constitutional treatment the author first names arsenic: Three drops of Fowler's Solution to be taken always after eating. Next, he mentions the Missisquoi water of St. Albans, Vermont, but says none have gained relief from the bottled waters—they must take it at the Springs.

Dr. F. A. Burral treated a case, with apparent cure, of cancer of the breast, with external applications of carbolic acid and glycerine, and internally sulph. quinia and carbolic acid. A tablespoonful of the solution of sulphate of quinia, (four grains to the ounce of water) with three drops of the solution of carbolic acid (two drops being equivalent to one grain of the crystals) was taken three times a day.

Such cases, to Dr. B., are of weight in sustaining the opinion of Mr. Moore, that the disease has a local origin, developing secondarily a blood contamination.

Locally, he thinks the knife preferable to the *ecraseur*, but that the operation is not to be thought of if the disease exist above the vaginal attachment of the cervix, or involve the mucous membrane of the vagina, or where there is any evidence of its existence in any other part of the body.

As to cauterizations he has strong conviction that they are often very useful in arresting the ulcerative process; in diminishing the profuse discharges, and thus saving the strength of the patient; in correcting the fetor, and thus lessening the chances of septic absorption. At an early stage the progress of the disease is manifestly retarded by their use. He employs principally the actual cautery, the acid nitrate of mercury and the chromic acid. When the disease has not extended to adjacent organs the actual cautery is most frequently applied. The acid nitrate in cases where granulations sprouting from the interior of the cervix show a tendency to bleed. This agent involves a strong tendency to salivation in broken-down cachectic habits, and is therefore often objectionable.

In cauliflower excrescence, where the disease has extended beyond reach of the knife, he applies carefully, with a camel's hair brush, solution of chromic acid (100 grains to the ounce of water.)

Usually this application does not involve pain, but one case is given where, six hours after, the most intense suffering ensued, requiring the persistent use of chloroform. From the report of Dr. Prouth, of London, he is disposed to try, when a proper case is presented, the bromic solution recommended—Bromine, gr. v.—x. to fifty minims of spirits of wine.

Among palliative measures, Dr. B. makes the reasonable proposition that the fear of making the patient an habitual opium eater, ought not to deter from prescribing that potent anodyne in this fearful disease. Belladonna, hyoscy. and cannab. indic., etc., are comparatively inefficient. Hydrate of chloral in full (30 gr.) doses, at bedtime, gives one of his patients a night of blissful sleep instead of distress and suffering.

In those cases where there is extreme sensitiveness of the ulcerated surface, pessaries of iodoform are useful.

| | | |
|---|---|---|
| ℞. | Iodoform, - - - - - - - - - - | gr. x. |
| | Butyri Cacao, - - - - - - - - - | ʒ j. |
| | Glycerine, - - - - - - - - - - | gtt. v. |

M. One pessary.

This also serves to correct fetor. A drachm of iodoform to the ounce of lard is also a useful ointment. As lotions to correct the odor, etc., solutions of bromine, permanganate of potash and carbolic acid are recommended—these to be carefully introduced so as thoroughly to wash the affected surface.

Want of space and time prevents further notice of this valuable paper.

*Braithwaite's Retrospect of Practical Medicine and Surgery.* Part LXI. July. Uniform American Edition. New York: W. A. Townsend & Adams, publishers. 1870. Pp. 299.

The current No. of Braithwaite is one of rather more than usual excellence, and of course will be procured as soon as its issue is made known, by every physician who has the least desire to keep pace with the profession.

*The Half Yearly Abstract of the Medical Sciences. Being a Digest of British and Continental Medicine, and of the Progress of Medicine and the Collateral Sciences.* Edited by William Domett Stone, M.D., F.R.C.S. (Exam.) vol. LI., July, 1870. Philadelphia: Henry C. Lea. Pp. 296.

*Reform, Medicine and Morals, No.* 3. An Address delivered before the Onondaga Medical Society, at Syracuse, N. Y., June 14, 1870. By J. A. Mowris, M.D.

A vehement attack on the utero vaginal specialty with all its paraphernalia of speculums, *porte caustiques*, pessaries, and gags. The writer characterizes this practice as the "speculoid" complaint, and charges the medical profession, by their indifference to it, as fostering one of the greatest medical absurdities of the age. We scissor a few paragraphs for specimens. Meanwhile we seriously apprehend the speaker has got himself into amazingly hot water.

"Basing a calculation on the *statements of the Uterine Specialists* as to the extent of their *trade* in a city of forty thousand, we discover in the uterus a susceptibility to derangement which may well astound the physiologist, alarm the friends of humanity, and impair veneration for Jehovah as the Maker of us all. The pretended revelations of the specialty teach that the "Great First Cause," in an attempt to create a harp of a thousand strings, exhausted itself on the last but one, and left the last to dangle at loose ends.

"But perhaps they proffer the convenient plea of 'new disease.' Very well. Nature then made a harp of a thousand strings, which went well enough for several thousand years, when with a revival of avarice, and the *advent of the speculum*, the harp fell hopelessly out of tune.

"The aggregate, *from the basis before mentioned*, is sufficient to supply *every woman in the city about three applications of the speculum per year*. Fifty per cent. of their number, six such operations; twenty-five per cent., twelve; twenty per cent., fifteen; ten per cent, thirty; and five per cent., *sixty operations*, which latter should certainly be enough to satisfy any speculator, and to cure the most obstinate case.

"We have thus disclosed to us an amazing prevalence of the 'peculiar' disease, or a remarkable number of patients undergoing a course of very diligent attention. Are all these really cases requiring treatment? Who believes it? Note the absurdity involved in the supposition. A like susceptibility to derangement in any *one* organ common to both sexes and all ages, would result, at once, in a grave popular calamity—an equal liability in *all* the other organs, would convert the world into a universal infirmary of helpless invalids.

Adam was the handiwork of God.
Was Eve the botch-work of a cobbler?

"Since this specialty has been courted by physicians hitherto reputable, it has been found convenient or necessary to defend it with a cool assumption of '*professional superiority*.' Professional superiority! The claim being prompted by an emergency, rather than by sheer vanity, it should not be too severely resented, but still, it deserves not to escape scrutiny. This vaunted superiority must consist in tact or scientific knowledge. Can it be that this superior tact of which they boast is all displayed in the application of the instrument. *Further*, the accounts of the specialists' performances rest so entirely on interested testimony, as to be quite apocryphal. That the uterus has been made by the specialist the subject of remarkable skill, is not favored by the law of probabilities; for, in attempting to suppose it, we are confronted by the query, whether physicians who have always shown a morbid solicitude to do their surgical skill where they may be seen of men, will be disposed to waste much of that precious commodity in secret."

But, positively, we dare not publish any more of this for fear that some of our friends will fancy they are our own sentiments, and their publication (facetiously) personal.

PAMPHLETS RECEIVED.

*Transactions of the Indiana State Medical Society.* 1870. Pp. 163. From the Secretary, G. V. Woolen, M.D., Indianapolis.

*Report to the Faculty of the Med. Dep. of the University of Louisiana in Regard to the Convention of Medical Teachers lately held in Washington City.*

*The Physical Exploration of the Rectum: With an Appendix on the Ligation of Hæmorrhoidal Tumors.* By William Bodenhamer, A.M., M.D. Illustrated by numerous drawings. New York: Wm. Wood & Co., 61 Walker street. 1870. Pp. 54.

*On the Origin of Diabetes, with some New Experiments Regarding the Glycogenic Functions of the Liver.* By W. T. Lusk, M.D., Prof. Physiology L. I. Med. College. New York: D. Appleton & Co. 1870. Pp. 19.

---

***Erotique Melancholy.***

From a treatise by James Ferrard, Dr. of Physick, published at Oxford in 1640, we extract:

"The regimen, or order of diet, in cure of love melancholy, differs not at all from that that is to be observed in the prevention of it, save only that it ought to be somewhat more humectative and less refrigerative; not forgetting, in the meane time, those meats that by some certaine occult properties they have in them, are found to be very good for those that are sick of this disease; as the turtle-dove, the heart of a wolfe, young owles taken and boyled in the juyce of marioram, the flesh of rats, and the like. And if the party be fallen away in his body, and is now grown very thin and dry, you must then prescribe him the same order of diet, according to Avicen, as you doe to those that are hecticall."

Dr. Ferrard does not recommend traveling in the cure, for "as one of the seaven Grecian sages said, change of place neither takes away folly nor teaches a man wisdom." Which is applicable to some other travelers also.

